# Cortisol Awakening Response Is Linked to Disease Course and Progression in Multiple Sclerosis

**DOI:** 10.1371/journal.pone.0060647

**Published:** 2013-04-16

**Authors:** Simone Kern, Ivonne Krause, Antje Horntrich, Katja Thomas, Julia Aderhold, Tjalf Ziemssen

**Affiliations:** Center for Clinical Neuroscience, MS Center, Department of Neurology, University Hospital Carl Gustav Carus at the Technische Universität Dresden, Dresden, Germany; University Hospital La Paz, Spain

## Abstract

**Objectives:**

Dysregulation of the hypothalamus-pituitary-adrenal (HPA) axis has frequently been reported in multiple sclerosis (MS). So far, HPA axis function in MS has predominantly been studied under pharmacological stimulation which is associated with a series of methodological caveats. Knowledge of circadian cortisol patterns and cortisol awakening response (CAR) is still limited.

**Methods:**

A total of 77 MS patients (55 relapsing-remitting MS (RRMS)/22 secondary-progressive MS (SPMS)) as well as 34 healthy control (HC) subjects were enrolled. Diurnal cortisol release was assessed by repeated salivary cortisol sampling. Neurological disability was rated by the Kurtzke’s Expanded Disability Status Scale (EDSS). Depressive symptoms and perceived stress were assessed by self-report measures.

**Results:**

RRMS but not SPMS patients differed in circadian cortisol release from HC subjects. Differences in cortisol release were restricted to CAR. Treated and treatment naïve RRMS patients did not differ in CAR. In a RRMS follow-up cohort (nine months follow-up), RRMS patients with EDSS progression (≥0.5) expressed a significantly greater CAR compared to HC subjects. RRMS patients with a stable EDSS did not differ from HC subjects. Neither depressive symptoms nor perceived stress ratings were associated with CAR in RRMS patients. In a step-wise regression analysis, EDSS at baseline and CAR were predictive of EDSS at follow-up (R^2^ = 67%) for RRMS patients.

**Conclusions:**

Circadian cortisol release, in particular CAR, shows a course specific pattern with most pronounced release in RRMS. There is also some evidence for greater CAR in RRMS patients with EDSS progression. As a consequence, CAR might be of predictive value in terms of neurological disability in RRMS patients. The possible role of neuroendocrine-immune interactions in MS pathogenesis is further discussed.

## Introduction

The cortisol awakening response (CAR) is a well described phenomenon which is characterized by a pronounced increase of cortisol within 20 to 30 minutes after awakening [1], [2]. While the precise mechanisms are still not entirely understood, CAR seems to be controlled by limbic regions [3], [4]. It is further modulated by various factors such as genetic polymorphisms [5], stressful experience [5], [6], [7], affective symptoms [6], [8] and inflammatory states [9]. Independently of the underlying modulating mechanisms, an elevated CAR indicates a hyperactive hypothalamus-pituitary-adrenal (HPA) axis with an increased diurnal cortisol release.

A hyperactive HPA axis has often been reported in multiple sclerosis (MS): in post mortem studies enlarged adrenals [10] as well as increased activity of corticotropin-releasing-hormone (CRH) producing cells within the hypothalamus have been found [11]. In response to intravenous administration of CRH, cortisol release was increased in MS patients compared to healthy control subjects [12], [13]. HPA axis function also seems to be linked to radiological as well as clinical aspects: increased cortisol response to CRH was associated with gadolinium enhancing lesions, a marker for acute central nervous system inflammation in MS [14]. In a three year follow-up study, increased adrenocorticotropic hormone (ACTH) response to CRH administration has been linked to disease progression and cognitive dysfunction [15]. A series of most recent studies indicates a complex interaction between depressive symptoms and diurnal cortisol release patterns in MS [16], [17].

So far, most insights into HPA axis function in MS are based on pharmacological and rather unphysiological interventions such as the administration of CRH and/or dexamethasone (Dex). Testing for HPA axis function under pharmacological conditions involves invasive methods (e.g. intravenous injections, repeated blood sampling) and is predominantly performed in a hospital setting. Both factors are capable of inducing a considerable amount of stress which by itself could possibly modulate cortisol release in response to the pharmacological challenge.

Knowledge of HPA axis function in MS under non-stimulated conditions is still limited [18] and data on diurnal cortisol release patterns including CAR in a non-hospitalized setting are rare [16], [17].

In the current study, we measured diurnal cortisol release including CAR under basal conditions in the absence of any pharmacological stimulation. Saliva cortisol sampling was the method of choice in order to allow for repeated, non-invasive sampling in patients’ home environment. We hypothesize that MS patients express an elevated diurnal cortisol release when compared to healthy control (HC) subjects. Relapsing-remitting (RRMS) as well as secondary-progressive (SPMS) MS patients were studied in order to identify a possible link between circadian cortisol release patterns and disease course as well as disease duration. Finally, we expected diurnal cortisol patterns to be associated with treatment [19], disease progression [15], affective symptoms [16], [17] and perceived stressful experience [6].

## Methods

### Participants

**Figure 1:**
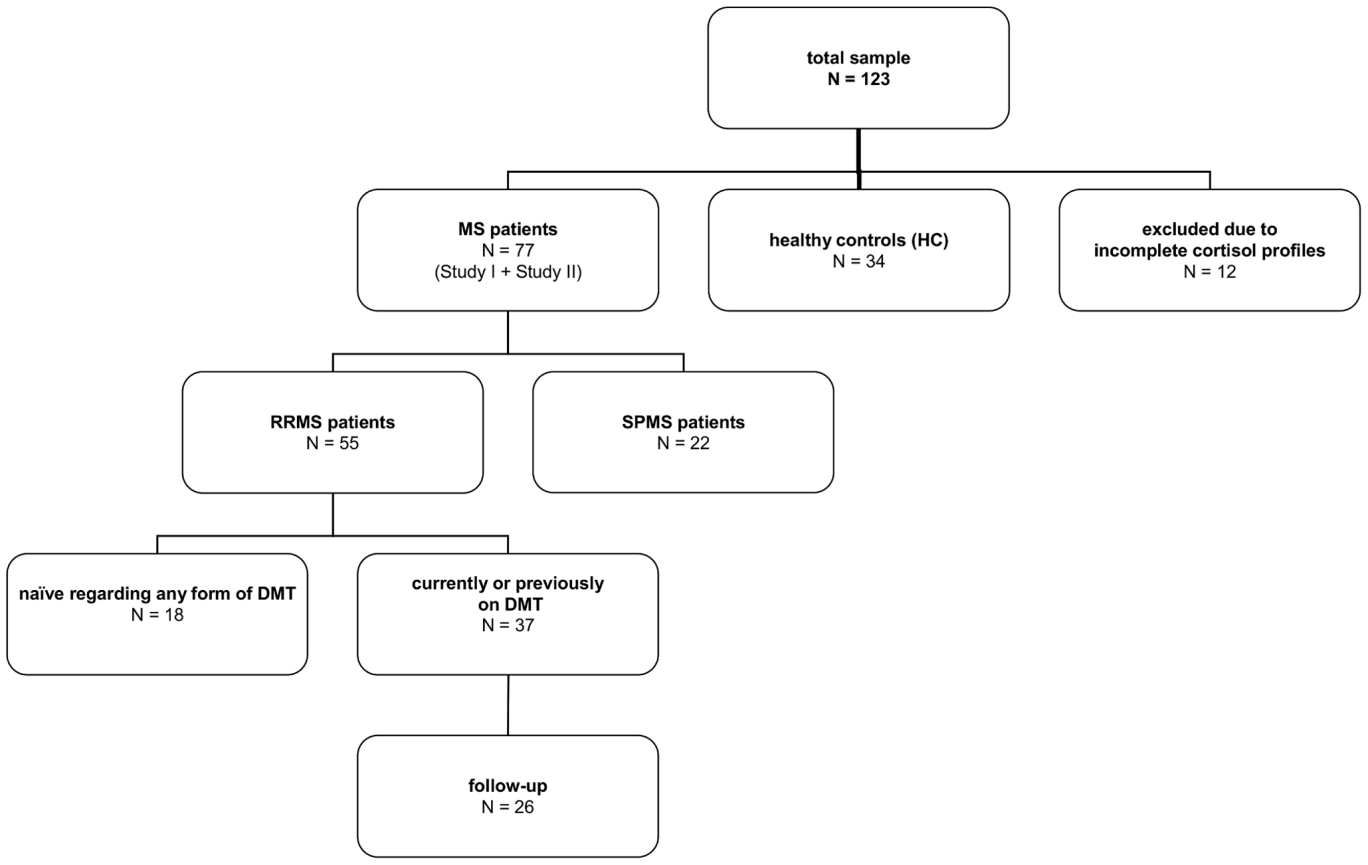
Sample description.

**Figure 2:**
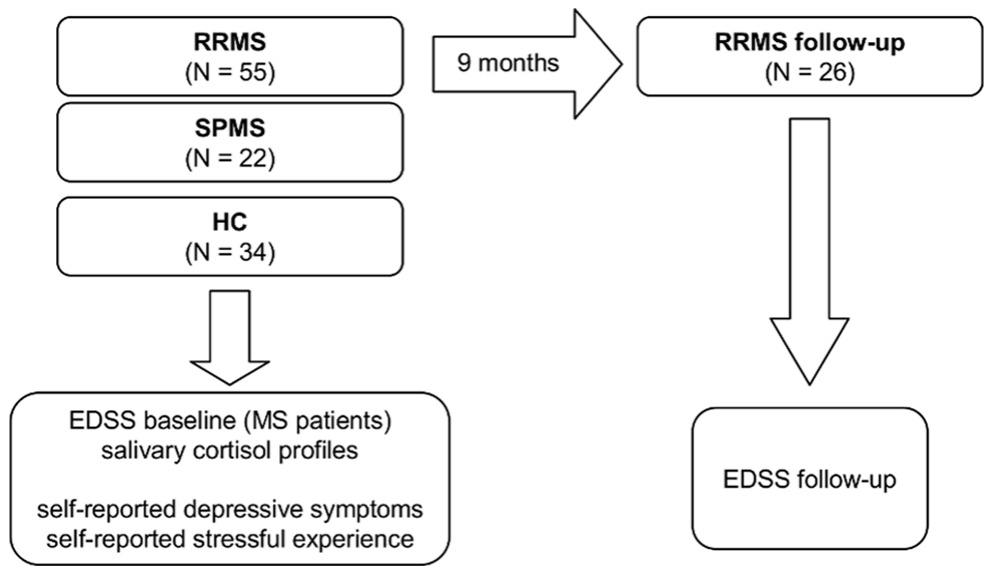
Study design.

Eighty-six patients with definite MS according to revised McDonald criteria [20] as well as thirty-seven HC subjects were enrolled. A total of twelve participants (nine MS and three HC subjects) had to be excluded from data analysis due to incomplete cortisol profiles. The final sample consisted of fifty-five RRMS patients, twenty-two SPMS patients and thirty-four HC subjects. At study entry, a total of eighteen RRMS patients had never received any form of MS disease modifying treatment (DMT) (RRMS naïve) so far. Twenty-six RRMS patients were studied over a follow-up period (RRMS follow-up) (figure 1 & 2). Characteristics of MS patients and HC subjects are listed in table 1.

**Table 1:** Characteristics for patients and healthy control (HC) subjects.

	RRMS total	RRMS currently or previously on DMT	RRMS naïve	RRMS follow-up total	RRMS follow-up with stable EDSS	RRMS follow-up with EDSS progression	SPMS	HC
**N**	55	37	18	26	17	9	22	34
**mean age**	36.56	36.59	36.50	40.08	39.65	40.89	45.91	35.56
(range)	(20–59)	(20–58)	(20–59)	(20–59)	(20–59)	(21–57)	(31–55)	(18–59)
**gender** (female/male)	31/24	21/16	10/8	14/12	8/9	6/3	15/7	19/15
**disease duration** 1	64.31	88.73	14.11	82.27	86.71	88.33	178.45	–
(range)	(0–325)	(1–325)	(0–123)	(1–325)	(1–290)	(10–325)	(11–316)	
**EDSS Baseline**	2.71	3.0	2.11	2.77	3.03	2.28	4.86	–
(range)	(1.0–6.0)	(1.5–6.0)	(1.0–3.5)	(1.5–5.0)	(1.5–5.0)	(1.5–4.5)	(2.0–7.0)	
**EDSS follow-up**	–	–	–	2.87	2.68	3.22	–	–
(range)				(1.5–5.5)	(1.5–5.0)	(2.0–5.5)		
**Difference in EDSS** 2	–	–	–	0.12	–0.32	0.94	–	–
(range)					(–1.0–0)	(0.5–2.0)		
**Interferon ß1a & 1b**		10	–	4	1	3	7	–
**Glatirameracetat**		7	–	6	5	1	3	–
**Natalizumab**		17	–	11	9	2	0	–
**Azathioprin**		1	–	1	1	0	0	–
**none**		2	–	4	1	3	12	–

1 time since diagnosis in months;

2 EDSS follow-up minus baseline).

### Neurological and Psychometric Measures

For MS patients, neurological impairment was rated using Kurtzke’s Expanded Disability Status Scale (EDSS) [21] based on the Neurostatus scoring system (www.neurostatus.net). EDSS ratings were performed by certified neurologists who have previously performed rater trainings to minimized inter-rater variability. Neurologists were blinded for information on HPA axis function and psychometric measures. Depressive symptoms were assessed by self-report measures (Center for Epidemiological Studies Depression (CES-D) Scale) [22], [23]. Perceived stress (past three months, screening scale) was assessed by the Trier Inventory for Chronic Stress (TICS) [24]. Self-report measures were received at baseline.

### Study Procedure

Participants were enrolled in two separate studies. Study I was a cross-sectional study which involved a single visit at the local MS centre. EDSS ratings were performed during this visit. Study II was a follow-up study which involved a total of four visits. For patients in study II, EDSS ratings were performed at the time of the initial visit as well as nine months after the initial visit. As a consequence, each MS patient received an EDSS rating at study entry (study I & study II). A total of twenty-six RRMS patients received two EDSS ratings over a period of on average nine months (mean 9.00 months/standard deviation (SD) 0.75) (study II). Please see figure 1 & 2 for illustration.

Exclusion criteria for MS patients (study I & II) were: inability (physical/cognitive) to follow the study procedure, age >60 years, steroid treatment within four weeks prior to study entry, pregnancy, acute or chronic bacterial/viral infection. Exclusion criteria for HC subjects (study I & II) were: MS diagnosis, a diagnosis of any other neurological or autoimmune disorder, age >60 years, steroid treatment, existence of any chronic inflammatory disorder, pregnancy, acute or chronic bacterial/viral infection.

Study protocols were reviewed and approved by the local ethics committee (Ethics Committee at the Technische Universität Dresden, Faculty of Medicine). All patients gave written informed consent prior to study entry.

### Cortisol Sampling

At the time of the initial visit, all participants were instructed to collect salivary cortisol samples on two separate days within a period of two weeks. Repeated sampling was performed in order to obtain a more reliable measure from each participant. Patients and HC subjects were equipped with two sets of Salivettes® (*Sarstedt, Nümbrecht, Germany*). Each participant was instructed to collect saliva samples on six points in time over the day at the home environment. In order to assess CAR, four measurements were taken within the first hour after awakening (awakening, 30 minutes, 45 minutes, and 60 minutes after awakening). Additional samples were collected at 3 pm and at 10 pm.

In order to avoid contamination with non-saliva fluid, solid particles or blood, all participants were instructed to refrain from eating, drinking or brushing their teeth prior to sampling. Patients on DMT were asked to keep an interval of 12 hours between the last DMT administration and saliva sampling. Collected samples were kept refrigerated at minus 20°Celsius until analysis. Cortisol concentrations were determined using a commercial luminescence immuno assay (LIA) (*IBL, Hamburg, Germany*) with an inter-assay coefficient of less than 5% and an intra-assay coefficient of less than 4%.

### Statistical Analysis

Statistical analysis was performed with Statistical Package for Social Sciences (SPSS (IBM), Version 19.0 for Windows).

In order to increase reliability, each participant’s average diurnal cortisol curve was calculated based on two separate profiles. For CAR (samples 1–4), an area under the curve with respect to ground (AUC_awakening_) was calculated as previously described [25].

Group differences for saliva cortisol values were analyzed using ANOVAs with repeated measures. Group differences for depressive symptoms, neurological disability, and AUC_awakening_ were analyzed using ANOVA with post-hoc tests (Bonferroni) or two tailed t-tests. Equal distribution of gender across groups was tested by the Chi^2^ test. Correlation analyses were performed with Pearson’s correlation.

## Results

### Circadian Cortisol Release and Disease Course

**Figure 3:**
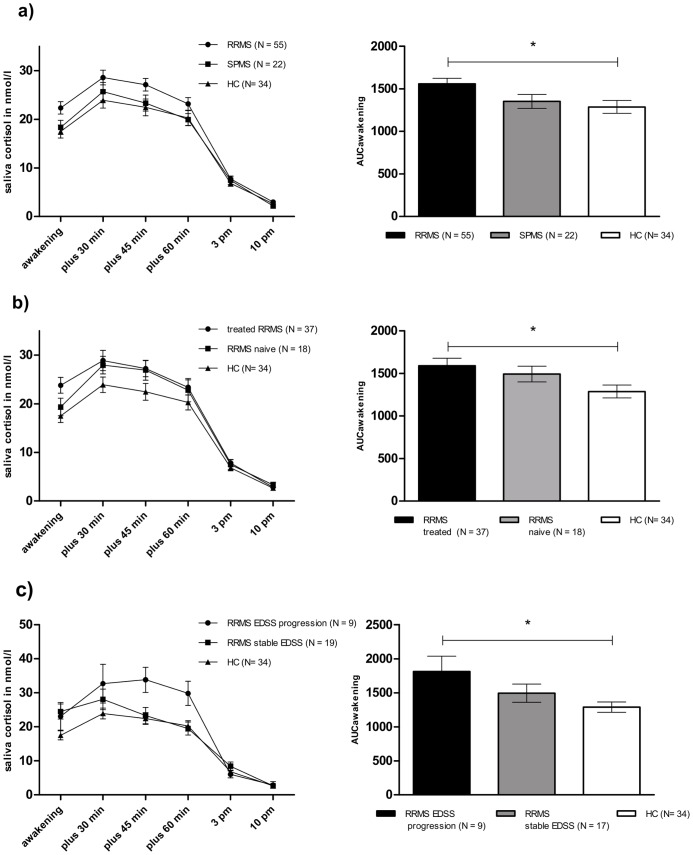
Mean circadian saliva cortisol and AUC_awakening_. a) RRMS/SPMS patients and HC subjects b) treated RRMS/treatment naive RRMS patients and HC subjects c) RRMS with stable EDSS/RRMS with EDSS progression ≥0.5 and HC subjects. Error bars reflect standard error of the mean. Significant results are marked (*reflects p<0.05).

Groups were sex-matched (*Chi^2^ test: p>0.05*) but SPMS patients were significantly older than RRMS patients (*ANOVA, post-hoc: p = 0.03*) and HC subjects (*ANOVA, post-hoc: p = 0.01*). Age was therefore considered as a covariate in the following analysis: Circadian cortisol release significantly differed between groups (*ANOVA with repeated measures: main effect group: F = 4.36, p = 0.015*) (figure 3a) and was most pronounced in RRMS patients (*age effect time × age: F = 0.65, p>0.05; main effect age: F = 0.18, p>0.05*). Differences in cortisol release between groups were restricted to CAR. Accordingly, RRMS patients showed a significantly greater AUC_awakening_ than HC subjects (*ANOVA, post-hoc: p = 0.023*). In contrast, SPMS patients did not differ in AUC_awakening_ from RRMS patients (*ANOVA, post-hoc: p>0.05*) or HC subjects (*ANOVA, post-hoc: p>0.05*) (figure 3a, table 2).

**Table 2:** Mean values and standard deviation for AUC_awakening_, self-reported depression and stress ratings (SD = standard deviation).

	RRMS total	RRMS curr. or previously on DMT	RRMS naïve	RRMS follow-up total	RRMS follow-up with stable EDSS	RRMS follow-up with EDSS progression	SPMS	HC
**N**	55	37	18	23	17	9	22	34
**AUC_awakening_**	1558.49	1590.57	1492.57	1603.78	1493.58	1811.93	1352.41	1288.30
(SD)	(492.16)	(536.05)	(392.49)	(602.21)	(549.17)	(674.96)	(380.12)	(441.38)
**depression ratings**	53.02	53.89	51.12	53.40	52.69	54.67	53.05	45.74
(SD)	(10.68)	(11.03)	(9.94)	(11.01)	(12.18)	(9.11)	(7.71)	(9.02)
**stress ratings**	50.83	50.68	51.19	51.13	51.63	50.13	52.77	45.35
(SD)	(10.79)	(9.40)	(13.83)	(9.47)	(10.28)	(8.15)	(7.67)	(10.39)

T-scores are listed for self-reported depression and stress ratings.

SPMS patients, but not RRMS patients showed a positive association between AUC_awakening_ and EDSS ratings (baseline) (table 3). AUC_awakening_ was not associated with disease duration in either group (table 3).

**Table 3:** This table lists correlations coefficients for the association between AUC_awakening_ on the one hand and EDSS (baseline & follow-up), disease duration, self-reported depressive symptoms and perceived stress (TICS screening scale) on the other hand.

Correlation for AUC_awakening_	RRMS total group	RRMS currently or previously on DMT	RRMS naïve	RRMS follow-up total	SPMS
**N**	55	37	18	26	22
**EDSS baseline**	0.09	0.12	−0.22	0.29	0.46*
**EDSS follow-up**	–	–	–	0.54**	–
**disease duration**	0.09	0.01	0.38	0.19	0.02
**depressive symptoms**	0.02	0.09	−0.24	0.11	−0.13
**stress ratings**	−0.02	0.09	−0.27	0.11	−0.48*

Correlation coefficients are displayed for RRMS patients (total group, RRMS naïve, RRMS follow-up) and SPMS patients. Pearson’s correlation coefficients are displayed. Significant results are marked (*p<0.05; **p≤0.01).

MS patients reported significantly more depressive symptoms than HC subjects (*ANOVA, post hoc: RRMS>HC, p = 0.003; SPMS>HC, p = 0.023*) while RRMS and SPMS patients did not differ in depressive symptom load (*ANOVA, post-hoc: p>0.05*) (table 2). Depressive symptoms were not associated with AUC_awakening_ in RRMS or SPMS patients (table 3). MS patients and HC subjects did not differ in perceived stress over the past three months (*ANOVA: p>0.05*) (table 2). Perceived stress was not associated with AUC_awakening_ in RRMS patients. However, in SPMS patients, AUC_awakening_ was associated with perceived stress (table 3).

### Circadian Cortisol Release in Treated vs. Treatment Naïve RRMS Patients

In order to test whether circadian cortisol release is modulated by DMT, we compared cortisol profiles from currently (or previously) treated and treatment naïve RRMS patients as well as HC subjects. Groups were matched with respect to age (*ANOVA: p>0.05*) and gender distribution (*Chi^2^ test: p>0.05*).

Circadian cortisol release significantly differed between groups (*ANOVA with repeated measures: main effect group: F = 3.58, p = 0.032)* (figure 3b). Differences were restricted to CAR. Accordingly, treated RRMS patients expressed a significantly greater AUC_awakening_ when compared to HC subjects (*ANOVA, post-hoc test: p = 0.027*). RRMS naïve patients did not significantly differ from treated RRMS patients or from HC subjects (*ANOVA, post-hoc: p>0.05*) (table 2, figure 3a).

Groups significantly differed in EDSS at baseline (*two-tailed t-test: treated RRMS>RRMS naïve, p = 0.003*) and disease duration (*two-tailed t-test: treated RRMS>RRMS naïve, p<0.001*). However, AUC_awakening_ was not associated with EDSS ratings or disease duration in either group (table 3). AUC_awakening_ was not associated with depressive symptoms or perceived stress in treated or treatment naïve RRMS patients (table 3).

### Circadian Cortisol Release in a RRMS Follow-up Cohort

EDSS progression was calculated based on the difference in EDSS at baseline and follow-up. Based on the EDSS difference, RRMS patients were divided into one progression group (EDSS progression ≥0.5) (N = 9) and into one stable group (EDSS progression ≤0) (N = 17) (table 1).

The two groups were matched according to age (*ANOVA: p>0.05*) and gender (*Chi^2^ test: p>0.05*) and did not differ in disease duration, EDSS at baseline or length of follow-up period (all *two-tailed t-test: p>0.05*). Although groups differed in frequency of immune modulating treatment (table 1), these differences were not statistically significant (*Chi^2^ = 9.57, p>0.05)*.

Over the follow-up period, EDSS ratings showed a significantly different pattern in the progression vs. non-progression group (*time × group interaction: F = 57.92, p<0.001*).

Groups significantly differed in terms of circadian cortisol release (*ANOVA with repeated measures: main effect group: F = 3.77, p = 0.029; time × group interaction: F = 3.42, p = 0.006*) (figure 3c). Differences were restricted to CAR and RRMS patients with an EDSS progression of ≥0.5 expressed a significantly more pronounced AUC_awakening_ than HC subjects (*ANOVA, post-hoc: p = 0.025*). RRMS patients with stable EDSS levels did not differ in AUC_awakening_ from HC subjects (*ANOVA, post-hoc: p>0.05*) (table 2, figure 3c). AUC_awakening_ was not associated with depressive symptoms or perceived stress in RRMS patients with stable or progressive EDSS (table 3).

Finally, we performed a stepwise linear regression analysis with EDSS at baseline and AUC_awakening_ as predictors for EDSS at follow-up.

Both variables significantly contributed to the model (*EDSS at baseline: ß = 0.65, p<0.001; AUC_awakening_: ß = 0.35, p = 0.011*) and explained 67% of the total variance (*R^2^ = 0.67*). In our model, EDSS at baseline explained 56% of variance. Adding AUC_awakening_ to the model explained for an additional 11%.

## Discussion

Although there have been several studies on HPA axis function in MS, to the best of our knowledge, this study is the first showing course specific differences in circadian HPA axis function including CAR.

Our data indicates that RRMS patients but not SPMS patients express a significantly greater CAR when compared to age and sex matched HC subjects. We were also able to show that these changes in CAR cannot be primarily explained by treatment effects or disease duration. Moreover, in a subgroup of RRMS patients, greater progression in neurological disability over a nine month follow-up period was associated with significantly greater CAR compared to HC subjects. We were also able to show, that the EDSS score at baseline in combination with CAR, was reasonably predictive of EDSS after nine months.

### Feedback Regulation versus Circadian HPA Axis Function

Elevated levels of ACTH and cortisol in response to CRH administration in MS patients have frequently been reported [13], [14], [26] and endocrine changes seem to be associated with clinical course as well as disease progression.

In a recent study, increased 24 hour free urinary cortisol as well as increased plasma morning cortisol levels (one time measurement between 8 am and 10 am) were shown in a relatively large cohort of MS patients [18]. HPA axis activity was most pronounced in RRMS patients during acute relapse. Clinically stable RRMS patients expressed intermediate levels while SPMS patients expressed only moderately elevated circadian cortisol levels [18]. Interestingly, patterns reported by Yssraelit and colleagues significantly differ from course specific patterns that are based on the Dex/CRH test [12] - an effect that could possibly be explained by methodological differences in HPA axis testing: while circadian regulation reflects an inherent release pattern that is most likely under the control of central structures such as the hippocampal formation [3], [4], the combined Dex/CRH test reflects an unphysiological stimulation protocol that was designed to test for feedback regulation on hypothalamic as well as pituitary levels. Thus, the Dex/CRH test and circadian cortisol levels reflect two very distinctively regulated aspects of the same endocrine system. In the light of the here presented data, we now have reason to believe, that in MS, circadian function and feedback regulation follow a unique course specific pattern in a way that feedback regulation is most disturbed in progressive disease courses such as SPMS and PPMS [12]. In contrast, circadian abnormalities seem to be less pronounced [18] or non-existent in SPMS patients. Here, RRMS patients, especially when in a clinically active state (e.g. acute relapse, progression in neurological disability), show most pronounced differences in circadian cortisol release. Interestingly, disease progression but not current EDSS or disease duration was associated with CAR in RRMS. This finding indicates that circadian cortisol response is predominantly affected by the acute disease state and not so much by previously accumulated deficits and disease duration per se.

### Treatment Effects and Circadian HPA Axis Function

Acute administration of Interferon-β (INF- β) has shown to cause significant changes in cortisol release within MS patients [19] and HC subjects [27]. It is therefore possible that HPA axis abnormalities seen in MS could be explained by treatment effects. What holds against this hypothesis is the fact that pronounced HPA axis abnormalities have been observed in a large group of untreated MS patients [18]. Also, HPA axis function seems to adapt to continuous INF-β administration in a way that cortisol levels normalize over the course of prolonged treatment (e.g. three months, one year) [19] [27]. In our study, treated RRMS patients and treatment naïve RRMS patients did not significantly differ in AUC_awakening_ which further supports the hypothesis that circadian HPA axis changes cannot be exclusively explained by treatment effects.

### Perceived Stress, Affective Symptoms and Circadian HPA Axis Function

An increased CAR [16] as well as increased evening cortisol levels [17] have been described in MS patients with depressive symptoms and in non-MS cohorts [28], [29]. In contrast to these previous findings, our data did not reveal such an association. RRMS and SPMS patients reported significantly more depressive symptoms compared to HC subjects but overall depressive ratings were comparatively low and not clinically significant. We therefore conclude that course specific circadian HPA axis differences exist in the absence of clinically relevant depressive symptoms.

However, in regard of previous findings [16], [17] we cannot rule out that more severe depressive symptoms potentially modulate circadian HPA axis function in MS patients.

MS patients (SPMS and RRMS) reported higher levels of perceived stress but mean T-scores were within a normal range and ratings did not significantly differ from HC subjects. Moreover, increased AUC_awakening_ in RRMS patients was not associated with perceived stress. Our data therefore indicate, that the observed differences in circadian HPA axis function in RRMS patients cannot be primarily explained by psychological factors such as perceived stress.

Interestingly, only in SPMS patients, AUC_awakening_ was positively associated with neurological disability and negatively associated with perceived stress, which indicates that circadian HPA axis function is more closely linked to neurological as well as psychological aspects in more advanced disease stages. Further studies are needed to elucidate the exact nature of this association.

### Limitations

Our study was not without limitations. While the overall sample size was quite reasonable, our follow-up cohort was comparatively small and EDSS progression was only studied over a relatively short period of nine months. As a consequence, the EDSS increase in the progression group was relatively small and could potentially be biased by inter-rater variability. Nonetheless, larger cohorts as well as longer follow-up periods seem necessary to further investigate the possible role of circadian HPA axis abnormalities in disease progression.

Overall depressive symptoms in our cohort where rather mild which could reflect a recruitment bias. Thus, further studies should specifically address circadian HPA axis function in subgroups of more severely depressed patients.

### Future Perspective on Neuroendocrine-immune Interaction in MS

The HPA axis and the immune system interact on multiple levels. It is a well- established finding, that a series of proinflammatory cytokines can activate the HPA axis [30]. Based on their findings, Besedovsky and del Rey suggested the existence of an immunoregulatory cytokine HPA axis circuit that plays an important role in the central control of systemic inflammation [30]. Within such a circuit, glucocorticoids (GC) have shown to inhibit the production of proinflammatory cytokines by inhibiting the nuclear transcription factor kappa B [31]. GC have also shown to influence a series of immunological functions that are associated with MS pathology such as Th1/Th2 balance [32], phospholipase A2 function [33] as well as the production reactive oxygen species [34]. MS is an inflammatory disorder and inflammation seems to be most pronounced during the relapsing-remitting course of the disease [35]. Accordingly, increased levels of proinflammatory cytokines have been reported in clinically active RRMS patients [18].

Increased circadian HPA axis activation in RRMS patients could therefore reflect a compensatory mechanism in response to increased central as well as systemic inflammation. This interpretation is in line with our finding that only RRMS patients with EDSS progression but not stable RRMS patients expressed a significantly greater CAR when compared to HC subjects. In order to test this hypothesis, further studies on circadian HPA axis function that also incorporate measures of GC effects on target tissues (e.g. GC sensitivity on immune cells) seem mandatory.
